# Comparison of community-acquired, hospital-acquired, and intensive care unit-acquired acute respiratory distress syndrome: a prospective observational cohort study

**DOI:** 10.1186/s13054-015-1096-1

**Published:** 2015-11-04

**Authors:** Kuo-Chin Kao, Han-Chung Hu, Meng-Jer Hsieh, Ying-Huang Tsai, Chung-Chi Huang

**Affiliations:** Departments of Thoracic Medicine, Chang Gung Memorial Hospital, Chang Gung University College of Medicine, (333) 5, Fu-Shing St., Kwei-Shan, Taoyuan Taiwan; Departments of Respiratory Therapy, Chang Gung Memorial Hospital, Chang Gung University College of Medicine, Taoyuan, Taiwan; Department of Respiratory Therapy, Chang-Gung University College of Medicine, Taoyuan, Taiwan; Departments of Pulmonary and Critical Care Medicine, Chang Gung Memorial Hospital, Chang Gung University College of Medicine, Chiayi, Taiwan

## Abstract

**Introduction:**

Acute respiratory distress syndrome (ARDS) is a syndrome characterized by diffuse pulmonary edema and severe hypoxemia that usually occurs after an injury such as sepsis, aspiration and pneumonia. Little is known about the relation between the setting where the syndrome developed and outcomes in ARDS patients.

**Methods:**

This is a 1-year prospective observational study conducted at a tertiary referred hospital. ARDS was defined by the Berlin criteria. Community-acquired ARDS, hospital-acquired ARDS and intensive care unit (ICU)-acquired ARDS were defined as ARDS occurring within 48 hours of hospital or ICU admission, more than 48 hours after hospital admission and ICU admission. The primary and secondary outcomes were short- and long- term mortality rates and ventilator-free and ICU-free days.

**Results:**

Of the 3002 patients screened, 296 patients had a diagnosis of ARDS, including 70 (23.7 %) with community-acquired ARDS, 83 (28 %) with hospital-acquired ARDS, and 143 (48.3 %) with ICU-acquired ARDS. The overall ICU mortality rate was not significantly different in mild, moderate and severe ARDS (50 %, 50 % and 56 %, *p* = 0.25). The baseline characteristics were similar other than lower rate of liver disease and metastatic malignancy in community-acquired ARDS than in hospital-acquired and ICU-acquired ARDS. A multiple logistic regression analysis indicated that age, sequential organ function assessment score and community-acquired ARDS were independently associated with hospital mortality. For community-acquired, hospital-acquired and ICU-acquired ARDS, ICU mortality rates were 37 % 61 % and 52 %; hospital mortality rates were 49 %, 74 % and 68 %. The ICU and hospital mortality rates of community-acquired ARDS were significantly lower than hospital-acquired and ICU-acquired ARDS (*p* = 0.001 and *p* = 0.001). The number of ventilator-free days was significantly lower in ICU-acquired ARDS than in community-acquired and hospital-acquired ARDS (11 ± 9, 16 ± 9, and 14 ± 10 days, *p* = 0.001). The number of ICU-free days was significantly higher in community-acquired ARDS than in hospital-acquired and ICU-acquired ARDS (8 ± 10, 4 ± 8, and 3 ± 6 days, *p* = 0.001).

**Conclusions:**

Community-acquired ARDS have lower short- and long-term mortality rates than hospital-acquired or ICU-acquired ARDS.

## Introduction

Acute respiratory distress syndrome (ARDS) is a significantly heterogeneous syndrome that involves many different groups of patients that may influence outcomes [1–3]. The Berlin definition classifies ARDS into mild, moderate, and severe by hypoxemia severity, using the PaO_2_/FiO_2_ ratio [4]. The predictive validity for mortality according to the Berlin definition has not been validated in recent studies [5–7]. The absolute predictive value is modest and suggests that some factors other than hypoxemia need to be investigated [8].

Differences in mortality rates have been demonstrated for patients with community-acquired pneumonia and hospital-acquired pneumonia [9]. For ARDS patients, a majority of patients developed acute lung injury (ALI)/ARDS within the first five days, especially 48–72 h after admission [10]. A retrospective cohort study showed a trend of decreasing prevalence in hospital-acquired and intensive care unit (ICU)-acquired ARDS patients over an eight-year period [11]. A prospective, multi-center, observational study revealed that late-onset ALI/ARDS patients had longer ICU and hospital stays than early-onset ALI/ARDS patients, but the mortality rate was not significantly different [12].

Little is known of the characteristics of patients in different ARDS subgroups according to the setting where the syndrome developed. The aim of this study is to investigate the outcomes of community-acquired, hospital-acquired and ICU-acquired ARDS patients. It has not been studied in the literature before and is a newly thought of patient population for ARDS. In addition to the severity of ARDS, the different categories may be an important factor for outcomes and, therefore, for clinical trials assessing the effects of potential interventions.

## Methods

### Study design and study population

A prospective observational study was conducted from September 2012 to August 2013 at Chang Gung Memorial Hospital, a tertiary care referral center with 3,700 ward beds and 278 adult ICU beds. These 278 adult ICU beds are distributed in 17 ICUs (nine medical ICUs, seven surgical ICUs, one burn ICU). All of the admitted patients with invasive mechanical ventilation were screened for eligibility by the Hospital Information System. Eligible patients were further evaluated when both chest-X-ray and PaO_2_/FiO_2_ ratio criteria were present concurrently. Patients were included if they met the criteria of the Berlin definition of ARDS and those with ARDS were further reviewed by a second independent investigator (LC Chiu) blind to the previous screening results. Differences were resolved by discussion between the two senior intensive care physicians (KC Kao and HC Hu).

Patients were excluded if they were younger than 18 years old or had been admitted to another hospital and referred for admission. The Chang Gung Memorial Hospital’s Institutional Review Board Ethics Committee approved the study protocol and informed consent was waived (CGMH IRB No.102-1729B).

### Definitions

The Berlin definition of ARDS includes: (1) onset within one week of a known clinical insult or new or worsening respiratory symptoms; (2) bilateral opacities not fully explained by effusions, lobar/lung collapse, or nodules; (3) respiratory failure not fully explained by cardiac failure or fluid overload and needs objective assessment (e.g., echocardiography) to exclude hydrostatic edema if no risk factor is present; and (4) PaO_2_/FiO_2_ ratio ≤300 mm Hg with positive-end expiratory pressure (PEEP) or continuous positive airway pressure (CPAP) ≥ 5 cm H_2_O [4].

According to the setting where the ARDS syndrome developed, we categorized ARDS patients into community-acquired, hospital-acquired and ICU-acquired ARDS. Community-acquired ARDS was defined as ARDS occurring upon admission or within 48 hours of hospital or ICU admission. Hospital-acquired ARDS was defined as ARDS occurring > 48 hours after hospital admission. ICU-acquired ARDS was defined as ARDS occurring > 48 hours after ICU admission (11). All the ARDS patients had the known clinical insults or worsening respiratory symptoms within one week.

### General management

It was recommended that patients be ventilated with protective ventilation using low tidal volume 4–8 mL/kg of predicted body weight plus moderate to high levels of PEEP for volume-controlled or pressure-controlled ventilation [13]. The predicted body weight of male patients was calculated as equal to 50 + 0.91 (height in centimeters - 152.4) and of female patients as equal to 45.5 + 0.91 (height in centimeters - 152.4). Ventilation was monitored by arterial blood gas measurements, with ventilator settings changed as needed. Pulse oximetry (SpO_2_) was used to monitor oxygenation and the FiO_2_ was adjusted to maintain SpO_2_ > 90 % or PaO_2_ > 60 mm Hg and to avoid raising the peak inspiratory pressure > 35 cm H_2_O.

The general medical management including fluid replacement, the use of antibiotics, corticosteroids in some selected patients and vasopressor agents, sedation with infusions of midazolam and paralysis with infusions of cisatracurium was directed by the intensivists-in-charge. The patients had a peripheral arterial line and PiCCO plus monitoring (version 5.2.2; Pulsion Medical System AG, Munich, Germany) for hemodynamic monitoring if indicated.

### Data collection

Demographics and baseline clinical information were collected on enrollment. Data on patient outcome 90 days after inclusion were tracked. The following data were recorded upon ICU admission: date of hospital and ICU admission, age, gender, body weight and height, underlying disease, and risk factors of ARDS. The mechanical ventilator settings (i.e., artery blood gas, tidal volume, lowest PaO_2_/FiO_2_ ratio with the highest PEEP, and peak airway pressure) were recorded during mechanical ventilation at the time of ARDS diagnosis. The Charlson comorbidity index (CCI) [14], Acute Physiology and Chronic Health Evaluation (APACHE) II score [15], Sequential Organ Failure Assessment (SOFA) score [16], Multiple Organ Dysfunction (MOD) score [17], and Lung Injury score (LIS) [18] were recorded on the day of inclusion and on days 3, 7, and 14 after inclusion. Regarding the definitions of liver disease in CCI, mild liver disease was chronic hepatitis or cirrhosis without portal hypertension. Moderate and severe liver diseases were cirrhosis combined with portal hypertension without and with history of varices bleeding [14].

The primary outcome was mortality (in ICU, at 28 days, at 60 days, and at 90 days, in hospital) and the secondary outcomes were ICU-free days and ventilator-free days. The number of ventilator-free days or ICU-free days was the mean number of days from day 1 to day 28 on which the patient had been breathing without assistance for at least 48 consecutive hours or which the patient had been transferred to ward from ICU. Patients who did not survive to 28 days were assigned zero ventilator-free days and zero ICU-free days.

### Statistical analysis

Descriptive statistics were expressed as mean ± SD (standard deviation). All variables were tested for normal distributions using the Kolmogorov-Smirnov test. Student t test was used to compare the means of continuous variables with normal distribution and the Mann–Whitney U test for the rest. Categorical data were compared using the chi square test. Risk factors for hospital mortality were analyzed using univariate analysis, and the variables statistically significant (p < 0.05) in the univariate analysis were included in the multivariate analysis by applying a multiple logistic regression based on backward elimination of data. The Hosmer-Lemeshow goodness-of-fit test was used for calibration when evaluating the number of observed and predicted deaths in risk groups for the death probabilities. Cumulative survival curves as a function of time were generated using the Kaplan-Meier approach and compared using the log-rank test. All statistical tests were two-tailed and *p* < 0.05 was considered statistically significant. All statistical analyses were performed using the SPSS (SPSS for Windows, SPSS Inc., Chicago, IL, USA) statistical package.

## Results

**Fig. 1:**
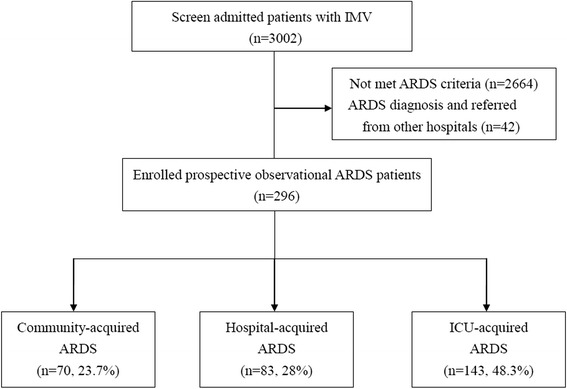
Flow chart for patients’ enrollment in the study. *IMV* invasive mechanical ventilation, *ARDS* acute respiratory distress syndrome

During the study period, 3,002 admitted patients with invasive mechanical ventilation were screened (Fig. 1). After excluding 2,664 patients who did not meet the ARDS criteria and 42 ARDS patients referred from other hospitals, 296 ARDS patients were included for analysis. Of these 296 ARDS patients, 24 patients underwent adjudication to have a definitive diagnosis of ARDS. The most common was ICU-acquired ARDS at 48.3 %, followed by hospital-acquired ARDS at 28 %, and community-acquired ARDS at23.7 %.

Demographic and clinical characteristics of the study patients revealed a male predominance (Table 1). The average lung injury score was 2.9 ± 0.5. The mean PaO_2_/FiO_2_ ratio was 129.1 ± 70.0 mm Hg. Following the Berlin definition, 51 patients (17.2 %) were classified as mild ARDS, 109 (36.8 %) as moderate ARDS, and 136 (46 %) as severe ARDS. The ICU mortality rates in mild, moderate, and severe ARDS patients were 50 %, 50 %, and 56 %, respectively, without a significant difference between the three groups (*p* = 0.25). Of the primary lung injury causes, pneumonia was the most common (n = 141), followed by aspiration (n = 52), inhalation injury (n = 4), and lung contusion (n = 2). Of the secondary lung injury causes, sepsis was the most common (n = 81), followed by major surgery (n = 8), acute pancreatitis (n = 6), and multiple transfusions with red blood cells (RBCs) (n = 2). Pressure-controlled mode was applied in 278 patients (93.9 %) and volume-controlled mode in 18 patients (6.1 %).

**Table 1:** Demographics and clinical characteristics of patients with different types of ARDS at the time of ARDS diagnosis

Characteristics	Total ARDS	Community-acquired ARDS	Hospital-acquired ARDS	ICU-acquired ARDS	*p*
	(*n* = 296)	(*n* = 70)	(*n* = 83)	(*n* = 143)	
Age (years)	63.0 ± 16.5	62.8 ± 19.9	62.3 ± 16.0	63.5 ± 15.2	0.855
Gender (male/female)	198/98	47/23	50/33	101/42	0.278
BMI (kg/m^2^)	23.6 ± 4.2	23.9 ± 3.9	23.6 ± 3.7	23.5 ± 4.6	0.811
PBW	57 ± 10	56.8 ± 8.0	56.2 ± 10.5	57.6 ± 10.6	0.580
CCI	3.4 ± 2.4	2.83 ± 2.2	3.63 ± 2.8	3.56 ± 2.2	0.068
APACHE II score	25.1 ± 6.9	26.1 ± 6.6	24.5 ± 6.9	25.0 ± 7.2	0.339
SOFA score	11.5 ± 3.8	10.7 ± 3.5	11.7 ± 4.0	11.8 ± 3.9	0.107
MOD score	9.8 ± 3.4	9.3 ± 2.9	9.9 ± 3.6	10.0 ± 3.6	0.300
Lung injury score	2.9 ± 0.5	3.1 ± 0.4	2.9 ± 0.5	3.0 ± 0.5	0.249
Tidal volume (ml/PBW)	8.4 ± 2.0	8.1 ± 1.7	8.7 ± 1.9	8.3 ± 2.1	0.241
PEEP (cm H_2_O)	10.2 ± 2.2	10.1 ± 2.0	9.8 ± 1.9	10.5 ± 2.5	0.078
Peak Paw (cm H_2_O)	29.8 ± 5.9	30.0 ± 5.2	29.6 ± 5.3	29.8 ± 6.5	0.918
pH	7.35 ± 0.11	7.33 ± 0.11	7.37 ± 0.12	7.35 ± 0.11	0.059
PaCO_2_ (mm Hg)	45.0 ± 17.3	44.9 ± 13.7	41.4 ± 14.9	47.2 ± 19.8	0.053
FiO_2_	0.8 ± 0.2	0.81 ± 0.19	0.79 ± 0.22	0.77 ± 0.23	0.082
PaO_2_/FiO_2_ (mm Hg)	129.1 ± 70.0	115.0 ± 66.4	132.3 ± 68.1	133.8 ± 72.3	
Severity of ARDS					
Mild	51(17.2 %)	9(12.9 %)	14(16.9 %)	28(19.6 %)	0.3383
Moderate	109(36.8 %)	24(34.3 %)	31(37.3 %)	54(37.8 %)	0.7677
Severe	136(46.0 %)	37(52.9 %)	38(45.8 %)	61(42.7 %)	0.6242
Cause of ARDS					
Pneumonia	141	34(48.6 %)	48(57.8 %)	59(41.3 %)	0.055
Sepsis	81	18(25.7 %)	21(25.3 %)	42(29.4 %)	0.755
Aspiration	52	11(15.7 %)	19(22.9 %)	22(15.9 %)	0.323
Major surgery	8	2(2.9 %)	3(3.6 %)	3(2.1 %)	0.733
Acute pancreatitis	6	1(1.4 %)	1(1.2 %)	4(2.8 %)	0.872
Others	8	2(2.9 %)	2(2.4 %)	4(2.8 %)	1.000
Components of CCI					
Myocardial infarct	10	1(1.4 %)	3(3.6 %)	6(4.2 %)	0.570
CHF	29	8(11.4 %)	9(10.8 %)	12(8.4 %)	0.729
Peripheral vascular disease	11	4(5.7 %)	2(2.4 %)	5(3.5 %)	0.550
Cerebrovascular disease	91	25(35.7 %)	24(28.9 %)	42(29.4 %)	0.562
Dementia	6	3(4.3 %)	1(1.2 %)	2(1.4 %)	0.306
Chronic pulmonary disease	31	11(15.7 %)	7(8.4 %)	13(9.1 %)	0.258
Connective tissue disease	1	0(0 %)	1(1.2 %)	0(0 %)	0.276
Ulcer disease	47	6(8.6 %)	13(15.7 %)	28(19.6 %)	0.118
Mild liver disease	12	1(1.4 %)	3(3.6 %)	8(5.6 %)	0.341
Diabetes without end organ damage	80	21(30 %)	17(20.5 %)	42(29.4 %)	0.284
Hemiplegia or paraplegia	16	5(7.1 %)	3(3.6 %)	8(5.6 %)	0.542
Moderate to severe renal disease	88	25(35.7 %)	18(21.7 %)	45(31.5 %)	0.137
Diabetes with end organ damage	3	0(0.0 %)	1(1.2 %)	2(1.4 %)	0.472
Any tumor without metastasis	44	8(11.4 %)	15(18.1 % )	21(14.7 %)	0.514
Leukemia	2	0(0 %)	0(0 %)	2(1.4 %)	0.341
Lymphoma	4	0(0 %)	1(1.2 %)	3(2.1 %)	0.616
Moderate to severe liver disease	47	0(0.0 %)	16(19.3 %)	31(21.7 %)	0.000^*^
Metastatic solid tumor	41	5(7.1 %)	17(20.5 %)	19(13.3 %)	0.057
AIDS	2	2(2.9 %)	0(0.0 %)	0(0.0 %)	0.039^*^

*Abbreviations*: *ARDS* acute respiratory distress syndrome, *ICU* intensive care unit, *BMI* body mass index, *PBW* predict body weight, *CCI* Charlson comorbidity index, *APACHE* acute physical and chronic health evaluation, *SOFA* sequential organ function assessment, *MOD* multiple organ dysfunction, *Paw* airway pressure, *PaO*_*2*_ */FiO*_*2*_ alveolar oxygen pressure/fraction of inspiratory oxygen, *PEEP* positive end expiratory pressure, *CHF* congestive heart failure, *AIDS* acquired immunodeficiency syndrome

All values are expressed as No. of patients (%) or mean ± SD

^*^ *p* < 0.05: Community-acquired ARDS vs. hospital-acquired ARDS vs. ICU-acquired ARDS

The demographic and clinical characteristics of different types of ARDS patients are shown in Table 1. Comparison among the three diagnosis groups revealed no statistically significant differences regarding age, gender, body weight, severity, causes, ventilation, oxygenation, and initial mechanical ventilation setting. Of the community-acquired ARDS patients, nine (12.9 %) were classified as mild ARDS, 24 (34.3 %) as moderate ARDS, and 37 (52.9 %) as severe ARDS. Of the hospital-acquired ARDS patients, 14 (16.9 %) were classified as mild, 31 (37.3 %) as moderate, and 38 (45.8 %) as severe ARDS. Of the ICU-acquired ARDS patients, 28 (19.6 %) were classified as mild, 54 (37.8 %) as moderate, and 61 (42.7 %) as severe ARDS. The distribution of severity in these three groups was not statistically significant (*p* = 0.65).

Prone position was applied to three patients with community-acquired ARDS, five with hospital-acquired ARDS, and three with ICU-acquired ARDS. Venovenous extra-corporeal membrane oxygenation was used in three patients with community-acquired ARDS, four with hospital-acquired ARDS, and three with ICU-acquired ARDS.

**Fig. 2:**
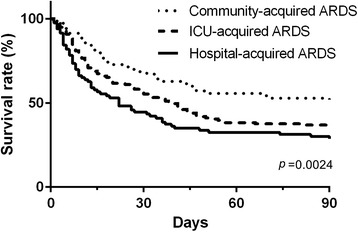
Kaplan-Meier survival curve of community-acquired ARDS, hospital-acquired ARDS, and ICU-acquired ARDS. *ARDS* acute respiratory distress syndrome, *ARDS* acute respiratory distress syndrome, *ICU* intensive care unit

In terms of outcome parameters, community-acquired ARDS patients had the lowest ICU mortality rate compared to hospital-acquired and ICU-acquired ARDS patients (37 %, 61 %, and 52 %, respectively, *p* = 0.001) (Table 2). The 28-day, 60-day, 90-day, and hospital mortality rates had the same trends. The overall survival rate in community-acquired ARDS was significantly higher than hospital-acquired and ICU-acquired ARDS (*p* = 0.0024) (Fig. 2). The number of ventilator-free days was significantly lower in the ICU-acquired ARDS patients than in the community-acquired and hospital-acquired ARDS patients (11 ± 9, 16 ± 9, and 14 ± 10 days, respectively, *p* = 0.001). The number of ICU-free days was significantly higher in the community-acquired ARDS patients than in the hospital-acquired or ICU-acquired ARDS patients (8 ± 10, 4 ± 8, and 3 ± 6 days, respectively, *p* = 0.001). Univariate analysis and multivariate logistic regression analysis were used to identify variables for hospital mortality that had significant prognostic value (Table 3). Identification of age [odds ratio 1.038, 95 % confidence interval (CI) 1.02-1.056, *p* < 0.001], SOFA score (odds ratio 1.287, 95 % CI 1.184-1.399, *p* < 0.001) and community-acquired ARDS (odds ratio 0.463, 95 % CI 0.250-0.855, *p* = 0.014) were significantly and independently associated with hospital mortality. Regression coefficients of these variables were used to calculate a natural logarithm of the odds (logit) of the probability of death (p), as follows: logit (p) = −5.206 + (0.038 × age) + (0.252 × SOFA score) + (1.029 × hospital-acquired ARDS) + (0.732 × ICU-acquired ARDS).

**Table 2:** Outcomes in different types ARDS patients

Outcomes	Community-acquired ARDS	Hospital-acquired ARDS	ICU-acquired ARDS	*p*
	(*n* = 70)	(*n* = 83)	(*n* = 143)	
Mortality –no.(% [95 % CI])				
In ICU	26 (37^*^[26–49])	51 (61[51–72])	75 (52[44–61])	0.001
At 28-day	31 (44^*^[33–56])	56 (68[57–78])	89 (62[54–70])	0.012
At 60-day	33 (47^*^[35–59])	58 (70[60–80])	92 (64[56–72])	0.001
At 90-day	33 (47^*^[35–59])	59 (71[61–81])	97 (68[60–76])	0.001
In hospital	34 (49^*^[37–60])	61 (74[64–83])	97 (68[60–76])	0.001
No. of ventilator-free days, days 1 to 28	16 ± 9	14 ± 10	11 ± 9^†^	0.001
No. of ICU-free days, Days 1 to 28	8 ± 10^*^	4 ± 8	3 ± 6	0.001

*Abbreviations*: *ARDS* acute respiratory distress syndrome, *MV* mechanical ventilation, *LOS* length of stay, *ICU* intensive care unit

All values are expressed as No of patients (% [95 % CI]) or mean ± SD

^*^ *p* < 0.05: Community-acquired ARDS vs. hospital-acquired ARDS and vs ICU-acquired ARDS

^†^ *p* < 0.05: ICU-acquired ARDS vs. Community-acquired ARDS and vs Hospital-acquired ARDS

**Table 3:** Univariate and multivariate logistic regression analysis of clinical variables associated with hospital mortality in ARDS patients

Parameter	Beta coefficient	Standard error	Odds ratio (95 % CI)	*p* value
Univariate logistic regression				
Age	0.024	0.008	1.024 (1.010-1.040)	0.001*
Female gender	−0.148	0.257	0.862 (0.522-1.426)	0.564
APACHEI II score	0.066	0.019	1.068 (1.029-1.108)	<0.001*
SOFA score	0.223	0.039	1.249 (1.157-1.349)	<0.001*
MOD score	0.175	0.041	1.191 (1.099-1.291)	<0.001*
PaO_2_/FiO_2_	−0.001	0.002	0.999 (0.996-1.003)	0.683
CCI	0.161	0.055	1.174(1.055-1.307)	0.003*
Moderate to severe liver disease	0.553	0.359	1.739 (0.860-3.516)	0.124
Metastatic solid tumor	0.929	0.415	2.532 (1.124-5.708)	0.025*
Community-acquired ARDS	−0.879	0.279	0.415(0.240-0.718)	0.002*
Multivariate logistic regression				
Age	0.037	0.009	1.038 (1.020-1.056)	<0.001*
SOFA score	0.252	0.043	1.287 (1.184-1.399)	<0.001*
Metastatic solid tumor	0.751	0.448	2.120 (0.881-5.099)	0.093
Community-acquired ARDS	−0.771	0.313	0.463 (0.250-0.855)	0.014*
Constant	−4.400	0.814	0.012	<0.001*

*Abbreviations*: *ARDS* acute respiratory distress syndrome, *CI* confidence interval, *APACHE* acute physical and chronic health evaluation, *SOFA* sequential organ function assessment, *MOD* multiple organ dysfunction, *PaO*_*2*_ */FiO*_*2*_ alveolar oxygen pressure/fraction of inspiratory oxygen, *CCI* Charlson comorbidity index

*p value < 0.05

The CCI was lower in community-acquired ARDS patients than hospital-acquired and ICU-acquired ARDS patients but the difference was not significant (2.83 ± 2.2, 3.63 ± 2.8, and 3.56 ± 2.2, respectively, *p* = 0.068) (Table 1). Regarding the components of CCI, community-acquired ARDS patients had less moderate to severe liver disease than hospital-acquired and ICU-acquired ARDS patients (0 %, 34 %, and 66 %, respectively, *p* = 0.000).

## Discussion

This one-year prospective observational study found that community-acquired ARDS patients had better short- and long-term mortality rates than hospital-acquired and ICU-acquired ARDS patients. Community-acquired ARDS patients had less underlying moderate to severe liver disease than hospital-acquired and ICU-acquired ARDS patients. The community-acquired ARDS patients had more ICU-free days than hospital-acquired or ICU-acquired ARDS patients and the ICU-acquired ARDS patients had fewer ventilator-free days than community-acquired or hospital-acquired ARDS patients.

The incidence of ARDS varies widely. Differences in demographics, healthcare systems, and definitions may account for different incidences of ARDS in different areas or countries. In adult population-based studies, the incidence of ARDS by the American-European consensus (AECC) definition ranged from 5–7.2 cases/100,000/year in Europe to 33.8/100,000/year in USA [11, 19–21]. For ICU patients, the reported incidence of ALI/ARDS by the AECC definition was 7.1 % in Europe and 7.7 % in Argentina [22, 23]. A cross-sectional study demonstrated that patients with ARDS correspond to about 5 % of hospitalized, mechanically-ventilated patients [24]. A recent study in Brazil revealed that the prevalence of ARDS by the Berlin definition in ICU patients was 1.8 % [6]. In this study, the incidence of ICU-acquired ARDS by the Berlin definition in mechanically-ventilated patients was 5.4 % (143/2,664), accounting for nearly 50 % of all three types of ARDS patients. Thus, the prevention of ARDS in ICU patients is an important issue. It is possible that a broad application of lung protective ventilation, better infection and aspiration control, and fewer blood transfusions can decrease the prevalence of ICU-acquired ARDS.

For critically-ill patients, the lead time of events is an important prognostic factor, but the correlation between the time of disease onset and outcomes is controversial. Late-onset septic shock (>24 h after ICU admission) was associated with a higher mortality rate but not significant in patients with early-onset septic shock (<24 h after ICU admission) (88 % vs. 63 %, *p* = 0.071) [25]. There was no difference in the mortality rates of patients with sepsis on ICU admission, those who developed sepsis within 48 h after ICU admission, and those who developed sepsis > 48 h after ICU admission (27 % vs. 20 % vs. 28 %, *p* = 0.526) in the Sepsis Occurrence in Acutely Ill Patients (SOAP) study [26]. However, in a multivariate analysis of patients with shock from the SOAP database, late-onset (>48 h after ICU admission) shock was an independent predictor of higher ICU mortality (odds ratio, 2.6; 95 % CI, 1.6-4.3; *p* < 0.001) [27]. For renal failure, later onset was associated with worse prognosis than early onset in ICU patients with mechanical ventilation [28–30]. For post-traumatic ARDS patients, there was no difference in mortality rates between the early (within 48 h of hospital admission) and late (>48 h of hospital admission) groups (27 % vs. 21 %) [31]. Sub-analysis of the SOAP database demonstrated no significant difference in ICU mortality rates between early- and late-onset ARDS (45.7 % vs. 35.5 %) [12].

The overall ICU mortality rate was not significantly different in mild, moderate, and severe ARDS (50 %, 50 %, and 56 %, *p* = 0.25) in this study. The previous study in ARDS patients found that the presence of serious comorbidities, such as acquired immune deficiency syndrome, metastatic cancer, immunocompromised, liver cirrhosis, and hepatic failure, almost tripled the mortality rate after hospital discharge compared to those with none [32]. The present study revealed that community-acquired ARDS patients had a significantly lower ICU mortality rate than hospital-acquired and ICU-acquired ARDS patients. Further analysis of the comorbidities showed that moderate to severe liver disease is less prevalent in the community-acquired ARDS patients than hospital-acquired and ICU-acquired ARDS patients (0 %, 19.3 %, and 21.7 %, *p* = 0.000). More patients with severe liver dysfunction may contribute to the high mortality rates in hospital-acquired and ICU-acquired ARDS patients than community-acquired ARDS patients. The patients with underlying severe chronic liver disease were always excluded in the most randomized controlled trials of ARDSnet [13, 33–37]. It is difficult to determine whether comorbidities with advanced liver disease or the severity of ARDS most influence the survival outcomes. The comorbidities could affect survival in ARDS patients and this needs further investigation.

For ARDS patients, there are many conditions associated with mortality. ARDS is a syndrome that is not, by itself, a cause of mortality but rather accompanies other disease processes. The cause of ARDS such as pneumonia, aspiration or sepsis, severity of ARDS, underlying comorbidities, and cause of death may relate to the mortality. In this study, the cause and severity of ARDS were not significantly different between the three categories. In addition to moderate to severe liver disease, more patients had underlying metastatic solid tumors in hospital-acquired and ICU-acquired ARDS than community-acquired ARDS. Given the poor prognosis of cancer patients with ARDS, it may explain, at least in part, the poor outcome in patients with hospital-acquired and ICU-acquired ARDS compared to community-acquired ARDS. Furthermore, it is possible that hospital-acquired and ICU-acquired ARDS may have worse prognosis due to the presence of hospital exposures that are known to increase the risk for hospital–acquired ARDS, such as aspiration, non-protective ventilation, inadequate antibiotics and source control, surgical or medical errors, and too much fluid and transfusion [38].

In this study, ICU-acquired ARDS patients had fewer ventilator-free days than the other two types of ARDS patients and had fewer ICU-free days than community-acquired patients (Table 2). These patients had already been treated in the ICU for more than two days due to previous insults or poor conditions before ARDS occurred. After suffering from ARDS, the longer ventilator use and ICU stay might be due to the fragile characteristics of these ICU patients. The medical cost in the ICU would be effectively reduced if we could recognize ARDS early and prevent its occurrence in these ICU patients.

Although some randomized controlled trials have reported improvements in mortality, the current overall hospital mortality is about 40-50 % in most series of ARDS patients [20, 39]. The different reported mortality rates in ARDS patients may be due to differences in patient selection, associated underlying diseases, predisposing or risk factors for ARDS, severity of hypoxemia, and setting of mechanical ventilation. For example, trauma-induced ARDS had favorable prognosis, with approximately 10 % 60-day mortality rate [40, 41]. However, the hospital mortality rate was higher at 68.8 % in cancer patients with ARDS [42]. In the present observational cohort study, the ICU mortality varies from 37 % to 61 % in three different types of ARDS. Other than the presence of malignancies and a high prevalence of liver failure, one of the reasons for the high mortality rate could be the use of high tidal volume (>9 ml/kg PBW) in a substantial proportion of this cohort (Table 1). As a consequence of high mortality and considerable variability in outcomes in ARDS patients, classification of phenotype for mortality is essential for predicting prognosis, guiding clinical decision-making, and designing prospective randomized controlled trials.

There are some limitations in this study. First, misdiagnosis of ARDS is a potential limitation derived from reliance on available diagnostic criteria. To diminish this possible bias, the diagnosis of ARDS was based on established criteria and the accuracy of diagnosis was verified through a separate case review by three independent intensive care physicians. Second, the study design limited patients to those who required invasive mechanical ventilation to identify those most at risk of subsequent mortality, thereby losing patients who met the criteria for ARDS but only received non-invasive ventilation. Third, a low tidal volume strategy was not fully applied in all of the ARDS patients and ventilator-induced lung injury might have contributed to mortality. However, this would have equally exerted an impact on all three types of ARDS patients and should not influence the results. Fourth, different management strategies may alter the ARDS-related outcomes, but this study does not identify the correlation between management strategies and mortality. Lastly, perhaps the main limitation is the single-center nature of the study population. Although strengthened by the prospective, cohort, and observational design, this may limit the generalization of the study results and warrant external validation.

## Conclusions

In this study, the overall ICU mortality rate was not significantly different in mild, moderate, and severe ARDS. According to the timing of onset and admission source, three types of patients with ARDS are classified as community-acquired, hospital-acquired, and ICU-acquired ARDS. The most common is ICU-acquired ARDS with 48.3 %, followed by hospital-acquired ARDS with 28 %, and community-acquired ARDS with 23.7 %. Community-acquired ARDS patients have better short- and long-term mortality rates than hospital-acquired or ICU-acquired ARDS patients. Underlying advanced liver disease may contribute to the different outcomes between these three groups. Patients with ICU-acquired ARDS have lower numbers of ventilator-free days and ICU-free days than those with community-acquired or hospital-acquired ARDS. These data provide relevant information on ARDS patients for evaluating individual outcomes and designing clinical trials.

## Key messages

- In this prospective study, according to the timing of onset and admission source, the most commonly occurring ARDS is ICU-acquired ARDS with 48.3 %, followed by hospital-acquired ARDS with 28 %, and community-acquired ARDS with 23.7 %.
- Community-acquired ARDS patients have better short- and long-term mortality rates than hospital-acquired or ICU-acquired ARDS patients.
- ICU-acquired ARDS patients have lower numbers of ventilator-free days and ICU-free days than those with community-acquired or hospital-acquired ARDS.
- In terms of different outcomes, classification according to the setting where the ARDS developed may be considered in designing clinical therapeutic trials in the future.

## Abbreviations

AECC: American-European consensus definition
ALI: Acute lung injury
APACHE: Acute Physiology and Chronic Health Evaluation
ARDS: Acute respiratory distress syndrome
CCI: Charlson comorbidity index
CPAP: Continuous positive airway pressure
ICU: Intensive care unit
LIS: Lung Injury score
MODS: Multiple Organ Dysfunction Score
PEEP: Positive-end expiratory pressure
SD: Standard deviation
SOAP: Sepsis Occurrence in Acutely Ill Patients
SOFA: Sequential Organ Failure Assessment
